# Where Are They Now? A Case Study of Health-related Web Site Attrition

**DOI:** 10.2196/jmir.4.2.e10

**Published:** 2002-11-22

**Authors:** Michael A Veronin

**Affiliations:** ^1^Texas Tech University Health Sciences Center School of PharmacyDepartment of Pharmaceutical SciencesTexasUSA

**Keywords:** World Wide Web, health-related Web sites, attrition, Internet Archive, Web site quality

## Abstract

**Background.:**

When considering health-related Web sites, issues of quality generally focus on Web content. Little concern has been given to attrition of Web sites or the "fleeting" nature of health information on the World Wide Web. Since Web sites may be available for an uncertain period of time, a Web page may not be a sound reference.

**Objective.:**

To address the issue of attrition, a defined set of health-related Web sites was examined at two separate time intervals.

**Methods.:**

To determine the degree of attrition, Web sites obtained and recorded from a previous study were revisited approximately three years later. From December 1998 to May 1999, 184 Web sites were collected from which health claims were identified. During May and June 2002, the previously recorded URL for each Web site was entered into the address field of the browser Netscape Navigator. It was documented whether the original Web site could not be found, moved to a different URL location, or the URL and site location was found unchanged from the original search. For a Web site whose URL remained unchanged, it was also noted whether the site had maintained currency, (i.e. updated) since the original posting. To ensure that inaccessibility may not be due to temporary server problems, another attempt was made to access the sites at different periods of time.

**Results.:**

When each URL address from the original set of 184 Web sites was re-entered into the address field of the browser, 108 (59%) of the sites could not be found, 31 (17%) had moved to a new URL address, and 45 (24%) of the sites could be found from the original URLs obtained in the previous study. Of the Web sites that moved to a new URL address, 7 sites provided a link from the original URL to redirect the viewer to the new location. Of the Web sites still in existence, 17 (38%) provided update information from the original posting.

**Conclusions.:**

It can be difficult to locate information that was previously found on the Web, and if a reference to an item is provided, there is no guarantee that viewers will be able to find the site at a later time. Enhancements in Web technologies such as the Internet Archive may improve this situation. Future research that is directed toward making sure Web site viewers know the site will be accessible at a later time will enhance the Web as a valuable medical information resource.

## Introduction

"We've all heard that a million monkeys banging on a million typewriters will eventually reproduce the entire works of Shakespeare. Now, thanks to the Internet, we know this is not true" [1].

Robert Wilensky

The results of a recent major national survey found that about 110 million people in the U.S. — over half of the adult population — may be seeking health information online [2]. This compares with 54 million in 1998, 69 million in 1999 and 97 million in 2001. And according to the American Medical Association, on any given day, more people go online for medical advice than actually visit health professionals [3].

When considering health-related Web sites, issues of quality generally focus on Web content: how to find relevant information, and how to assess the credibility of the publisher as well as the accuracy and reliability of a document retrieved [4]. Little concern has been given to attrition of Web sites or the "fleeting" nature of health information on the Web.

Scientist and scholar Sir Isaac Newton once said, "If I have seen farther than others, it is because I was standing on the shoulders of giants" [5]. Much, if not all, scholarship is based on relation to previous work, and when new scholarly work is produced, it is important that detailed and accurate information on sources consulted are cited. To facilitate referencing, scholarly works have been routinely collected and preserved in print by libraries and database producers [6,7]. But in terms of cataloging, storage and retrieval as it relates to the Web, the status quo does not apply. With the advent of the Web, libraries must now consider Web site information that may be created, change, move, expire and disappear; with no record of the information being preserved. Few libraries made the practice of collecting copies of Web pages [8].

Since Web sites may be available for an uncertain period of time, a Web page may not be a sound reference. If a Web page or link disappears, chances are almost nonexistent of locating the reference at a later time. As a safeguard, it has been recommended that individuals keep a personal copy of Web pages as evidence that the information existed [9].

To address the issue of attrition, a defined set of health-related Web sites was examined at two separate time intervals.

## Methods

In an earlier study, a systematic survey was conducted to determine the validity of health claims on the World Wide Web for the herbal remedy *Opuntia* [10]. From December 1998 to May 1999, 184 Web sites were collected from which health claims were identified. Web sites were retrieved utilizing multiple search engines, and the Uniform Resource Locator (URL) for each Web site was recorded.

In this study, to determine the degree of attrition, each of the 184 Web sites obtained and recorded from the previous study were revisited at a later period of time. During May 2002, the previously recorded URL for each Web site was entered into the address field of the browser Netscape Navigator (version 4.7, Netscape Communication Corporation, Mountain View, California.) It was documented whether the original Web site could not be found, moved to a different URL location, or the URL and site location was found unchanged from the original search. For A Web site whose URL remained unchanged, it was also noted whether the Web site had maintained currency, (i.e. updated) since the original posting.

Since it is conceivable that inaccessibility of Web sites may be due to temporary server problems, another attempt was made to access the sites at different periods of time. For each "HTTP Error 404" or similar message obtained from the initial URL checks, an attempt to access these sites was made during June 2002 on various days and times of day in the manner described above.

## Results

Results indicate that when each URL address from the original set of 184 Web sites was re-entered into the address field of the browser, 108 (59%) of the sites could not be found, 31 (17%) had moved to a new URL address, and 45 (24%) of the sites could be found from the original URLs obtained in the previous study. Of the Web sites that moved to a new URL address, only 7 sites provided a link from the original URL to redirect the viewer to the new location. Of the Web sites still in existence, 17 (38%) provided update information from the original posting. The information is summarized in Table 1.

**Table 1:** Attrition of Health-related Web Sites for a Three-year Period **

Web Site Sponsor (No. of Sites)	Not Found	Moved To New URL	URL Redirected	URL to Site as Original	Maintenance Update Provided
Herbal Vendor (74)	46	14	1	14	7
Food/Recipes Products (7)	5	1	0	1	0
Educational Institution(24)	12	1	0	11	6
Government Institution (3)	1	2	1	0	1
Historical Essay (8)	1	1	1	6	0
Travel and Tourism (5)	1	2	0	2	1
Message Board (15)	15	0	0	0	0
Reference Guide (16)	8	6	3	2	1
Print Media* (24)	17	2	1	5	1
Expert (7)	2	2	0	3	0
Doomsday Group (1)	0	0	0	1	0
Totals (184)	108 (59%)	31(17%)	7(4%)	45 (24%)	17 (38%)

^*^ Includes book excerpts, newspaper and magazine articles, newsletters, a calendar reprint and a radio broadcast transcript

^**^ Original Web site addresses and content are available on the World Wide Web at http://ismo.ama.ttuhsc.edu/users/~veronin/WebOpuntia.pdf

In this study, attrition is defined as the unavailability of a Web site when known to be previously accessible based on a known URL address. This did not include sites that were redirected to a new URL.

Approximately three years after initial posting, over two-thirds of the health-related Web sites reviewed could not be found or had moved with no forwarding URL, and about one-third of the remaining sites maintained currency of information. It appears that links are terminated as Web sites are moved or removed, or as servers close down. This supports the notion that it is difficult, if not impossible, to locate information that was previously found on the Web, and if a reference to an item is provided, there is no guarantee that viewers will be able to find the site at a later date.

In this study, a comprehensive data set of Web sites on a specific health-related topic was obtained, and attrition was examined. Obviously an example from a single health-related topic is limited in what conclusions should be drawn. These findings cannot be generalized to other medical topics. But this raises the question that other health-related sites on the World Wide Web may vary in their degree of attrition, and warrants further research into methods of dealing with attrition with other medical topics.

## Discussion

The average life of a Web page is about 77 days [11]. The perceived value of the Web lies in the immediate accessibility to a seemingly endless pool of information with no central controlling authority. This also makes the Web difficult to maintain. According to Chris Sherman, Associate Editor of SearchEngineWatch.com, (http://searchenginewatch.com),as automatic maintenance, most search engines remove missing URLs from their index when they recrawl and find that the pages are gone [12]. A different problem arises, though, when an organization has gone out of business but its site still exists. This is a much more difficult problem to handle, and to date, no search engine exists to locate or remove these sites.

Enhancements in Web technologies hope to improve the problem of attrition. A prime example is the Internet Archive.

### The Internet Archive

The Internet Archive (http://www.archive.org) is a digitallibrary of Web pages created with the lofty goal of cataloging all of the past and present publicly available material on the World Wide Web [11]. Accessible to the public for free, it contains more than 100 terabytes of data and is growing by 10 to 12 terabytes a month. Since 1996, the Internet Archive has been storing Web pages, including graphics files, from publicly accessible Web sites. A feature implemented October 2001 known as the "Wayback Machine" allows users to go back and view earlier versions of current Web sites or of Web sites that no longer exist.

The Wayback Machine serves as a source to find Web pages when the page or host cannot be located [11]. When a user encounters a "File Not Found" or similar message on the Web, the Wayback Machine can be accessed to find a facsimile of the Web page.

Though a significant accomplishment towards recovering lost Web pages, the Wayback Machine has limitations. It is not searchable by keywords or text in the manner of a general search engine. The user must know the precise URL of a particular Web page or site to access the Archive. Having entered a URL address, the viewer is presented with a list of dates that designates when a particular page was archived. Also, though the Internet Archive contains more than 100 terabytes of data, much is still missing. For example, it does not contain the older gopher content and other non-Web files prior to 1996, and a relatively small number of pages exists from 1996, with content increasing to recent times.

### Issues of Quality and Content

The question may arise as to whether a relationship exists between Web site quality and attrition. Are poor quality sites more likely to disappear in time than sites of higher quality?

A consensus has yet to be reached as to the properties a Web site needs to have to be considered "high quality." Wilson states that "quality remains an inherently subjective assessment, which depends on the type of information needed, the type of information searched for, and the particular qualities and prejudices of the consumer" [13]. Yet many organizations and individuals have identified standards of quality that should be applied to the Web [14]. A practical approach for assessment has been described by Risk that provides benchmarks of quality [15]. It includes assessing a site for information that is accurate, current, has a clear source, is referenced, has disclaimers and cautions if appropriate, clear, clean and pleasing design features and a well-defined purpose. These criteria were applied to the original sites in this study by this author to examine whether attrition may be influenced by quality. If a site possessed at least 5 of these attributes, it was considered "high" quality, 3 to 4 attributes, it was considered "moderate," and 2 or less it was considered "poor" quality. The results are summarized in Table 2.

It appears that although the high quality sites make up only a small portion of the total number of sites retrieved (15%), half of the original high quality sites (14 of 28) could be located from the original URL or were redirected to a new URL. Conversely, only 10 of the 73 poor quality sites were accessible from the original URL entry, and only one poor quality site was redirected to another URL from the original site. This suggests that Web sites of higher quality may be less subject to attrition than those of poorer quality, and warrants further research on the relationship between Web site quality and attrition with other medical topics.

Considering subject matter and attrition, it may be that certain topics (such as herbal remedies) can have periods of enthusiasm by the public then wane — which may be the case with these sites. Perhaps information on more mainstream topics (such as health risks and smoking) is less vulnerable to attrition.

**Table 2:** Quality of Health-related Web Sites and Attrition

Web Site Quality* (No. of Sites)	Not Found	Moved To New URL	URL Redirected	URL to Site as Original
High (28)	10	7	3	11
Moderate (83)	38	21	3	24
Poor (73)	60	3	1	10
Totals (184)	108 (59%)	31 (17%)	7(4%)	45 (24%)

^*^ Quality assessed by author based on attributes described by Risk [15]: High = 5 or more, Moderate = 3 to 4, Poor = 2 or less

### Future Considerations

It has yet to be determined with certainty the forces that influence the survival of Web sites. With the complex and dynamic nature of information flow on the Web, is there a form of "natural selection" at work in health Web site survival? If attrition is not related to the site's quality or subject matter, perhaps those with strong commercial backing may survive with greatest frequency. At this point we can only speculate what will endure.

In some instances, Web site attrition may be desirable. A common complaint against search engines is that they return too many pages, and that many of the pages have low relevance to the query [16]. The most efficient search engines index only a fraction of the total number of documents on the Web, [17] and if sites of poorer quality go away, ideally this should help retrieval of documents of higher relevance to the user.

Most quality issues with the Web focus on consumers, [18] however, a recent major poll revealed that physicians are using the Internet to increase their medical knowledge and improve the care they provide to patients [19]. Medical information can change rapidly with continuing breakthroughs and advances in medical knowledge. Availability of information through the Web would facilitate access to the most up-to-date information on current medical topics and scientific discoveries. Future research that is directed toward making sure Web site viewers always know the site will be accessible at a later time will enhance the Web as a valuable medical information resource.
